# Low-Power Laser Irradiation Suppresses Inflammatory Response of Human Adipose-Derived Stem Cells by Modulating Intracellular Cyclic AMP Level and NF-κB Activity

**DOI:** 10.1371/journal.pone.0054067

**Published:** 2013-01-16

**Authors:** Jyun-Yi Wu, Chia-Hsin Chen, Chau-Zen Wang, Mei-Ling Ho, Ming-Long Yeh, Yan-Hsiung Wang

**Affiliations:** 1 Institute of Biomedical Engineering, National Cheng Kung University, Tainan, Taiwan, Republic of China; 2 Department of Physical Medicine and Rehabilitation, Kaohsiung Medical University Hospital, Kaohsiung, Taiwan, Republic of China; 3 Department of Physical Medicine and Rehabilitation, Faculty of Medicine, College of Medicine, Kaohsiung Medical University, Kaohsiung, Taiwan, Republic of China; 4 Department of Physical Medicine and Rehabilitation, Kaohsiung Municipal Ta-Tung Hospital, Kaohsiung, Taiwan, Republic of China; 5 Orthopaedic Research Center, College of Medicine, Kaohsiung Medical University, Kaohsiung, Taiwan, Republic of China; 6 Department of Physiology, College of Medicine, Kaohsiung Medical University, Kaohsiung, Taiwan, Republic of China; 7 Graduate Institute of Medicine, College of Medicine, Kaohsiung Medical University, Kaohsiung, Taiwan, Republic of China; 8 School of Dentistry, College of Dental Medicine, Kaohsiung Medical University, Kaohsiung, Taiwan, Republic of China; University of Iowa Carver College of Medicine, United States of America

## Abstract

Mesenchymal stem cell (MSC)-based tissue regeneration is a promising therapeutic strategy for treating damaged tissues. However, the inflammatory microenvironment that exists at a local injury site might restrict reconstruction. Low-power laser irradiation (LPLI) has been widely applied to retard the inflammatory reaction. The purpose of this study was to investigate the anti-inflammatory effect of LPLI on human adipose-derived stem cells (hADSCs) in an inflammatory environment. We showed that the hADSCs expressed Toll-like Receptors (TLR) 1, TLR2, TLR3, TLR4, and TLR6 and that lipopolysaccharide (LPS) significantly induced the production of pro-inflammatory cytokines (Cyclooxygenase-2 (Cox-2), Interleukin-1β (IL-1β), Interleukin-6 (IL-6), and Interleukin-8 (IL-8)). LPLI markedly inhibited LPS-induced, pro-inflammatory cytokine expression at an optimal dose of 8 J/cm^2^. The inhibitory effect triggered by LPLI might occur through an increase in the intracellular level of cyclic AMP (cAMP), which acts to down-regulate nuclear factor kappa B (NF-κB) transcriptional activity. These data collectively provide insight for further investigations of the potential application of anti-inflammatory treatment followed by stem cell therapy.

## Introduction

Adult stem cells isolated from fat tissue, or adipose-derived stem cells (ADSCs), are capable of differentiating into bone, cartilage, fat, and muscle in vitro [1] and are a potential source of mesenchymal stem cells (MSCs). The advantages of ADSCs comparing to bone marrow and embryotic stem cells include the fact that they are less invasive when used in operations, can be safely transplanted to an autologous or an allogeneic host, and exhibit increased stem cell activities [2]. A variety of studies involving animal models, preclinical trials, and clinical trials have suggested that MSC-based tissue regeneration provides beneficial effects with respect to replacing damaged tissues and restoring organ function [3]–[6]. Although the mechanism underlying this type of regeneration is still not well understood, stem cell regeneration has been postulated to relate to the survival rate of engrafted cells [7] and the microenvironment of the local injury site. During tissue repair, inflammation represents an important phase in the healing process resulting in an influx of neutrophils/macrophages and release of pro-inflammatory cytokines and growth factors to remodel injury sites [8]. The existence of inflammatory mediators is likely restrictive regarding the applicability of stem cell-based implantation [9].

Lipopolysaccharide (LPS), the ligand of Toll-like receptor (TLR) 4, is a common model system that has been used in several recent studies to investigate inflammatory reactions. Toll-like receptors (TLRs) are characterized as transmembrane proteins composed of a globular intracellular region and an extracellular domain that contains numerous leucine-rich repeats (LRR) [10]. Ligation through TLRs leads to the recruitment of TIR-domain-containing adaptor proteins (MyD88, TRIF, and TIRAP) that trigger the activation of a nuclear factor kappa B (NF-κB) signaling cascade [11], [12]. As a result of this activation, transcription factor NF-κB induces the secretion of pro-inflammatory cytokines, including TNF-α, IL-1β, IFN-γ, and IL-6, thereby promoting the immune response.

Over the last few decades, low-power laser irradiation (LPLI) has been applied clinically for treating musculoskeletal aches and pains, wound healing, chronic inflammation, and acute inflammation. Several studies have suggested benefits of using LPLI. For example, Bortone et al. noted that LPLI induced an anti-inflammatory effect through the regulation of transcription factors linked to Cox-2 and through regulating the expression of pro-inflammatory mRNAs [13]. Additionally, Aimbire et al. reported that LPLI reduced neutrophil influx and IL-1β mRNA expression [14], and Sakurai et al. showed that LPLI inhibited PGE2 production and led to a reduction of Cox-2 mRNA levels [15]. However, few studies have been performed to investigate the effect of LPLI on the LPS-induced inflammatory response of ADSCs. The purpose of this study was to evaluate the effect of LPLI on the LPS-induced inflammation response of hADSCs and to provide basic knowledge related to the clinical application of stem cell therapy.

## Materials and Methods

### Cell Culture

The hADSCs used in this research was purchased from Cellular Engineering Technologies (CET, Coralville, IA, USA). The hADSCs were cultured and expanded in Keratinocyte serum-free medium (KSFM, GIBCO-BRL, Rockville, MD, USA) containing the supplements bovine pituitary extract (25 mg) and Human Recombinant EGF protein (2.5 μg) as well as 0.2 mM N-acetyl-Cysteine (Sigma-Aldrich, Saint Louis, MO, USA), 0.2 mM L-Ascorbic acid 2-phosphate magnesium (Sigma-Aldrich, Saint Louis, MO, USA), 5% fetal bovine serum, and 1% penicillin/streptomycin (GIBCO-BRL, Rockville, MD, USA) and a mixture of Dulbecco’s modified Eagle’s medium-low glucose (GIBCO-BRL, Rockville, MD, USA) containing 0.06 μg/ml insulin (Sigma-Aldrich, Saint Louis, MO, USA), 5% fetal bovine serum, and 1% penicillin/streptomycin in a ratio of 1∶1. The cells were cultured in a humidified 5% CO_2_ atmosphere in a 37°C incubator, and the medium was changed every other day.

The U937 human leukemic monocyte lymphoma cell line was obtained from the American Type Culture Collection (a kind gift from Dr. Chi-Ming Chiu, Department of Biotechnology, Ming Chuan University, Taipei, Taiwan). Growing U937 cells were cultured using RPMI medium 1640 (GIBCO-BRL, Rockville, MD, USA) with L-glutamine, 10% fetal bovine serum and 1% penicillin/streptomycin; the cells were maintained at 37°C in an atmosphere of 5% CO_2_. Fresh medium was added two times a week, and the cells were kept at a density between 2×l0^5^ and 8×l0^5^ cells/ml.

### Low-power Laser Irradiation

A red gallium-aluminum-arsenide (GaAlAs) laser (wavelength 660 nm) (TRANSVERSE IND. CO., LTD., Taipei, Taiwan) was used as a light source follow our previous study [16]. The laser had a maximum power of 70 mW, and the distance between the laser source and the bottom of the plate could be adjusted to match the intended target size. The power density applied was 15.17 mW/cm^2^, and the cells were irradiated for 264 s and 528 s to achieve energies of 4 J/cm^2^ and 8 J/cm^2^, respectively. All irradiation experiments were performed on a clean bench at room temperature. The control groups were processed under the same conditions, except without laser irradiation.

### Chemical Reagent Treatment

LPS (Sigma-Aldrich, Saint Louis, MO, USA) was dissolved in 1X phosphate-buffered saline (PBS, Bio-Tech, Santa Cruz, CA, USA) to a final concentration of 10 mg/ml and then stored at 4°C. The LPS concentrations used in this study were 10, 50, 100, and 500 ng/ml; LPS was freshly diluted in culture medium immediately before being used. Forskolin (Sigma-Aldrich, Saint Louis, MO, USA), an adenylyl cyclase activator, was used as a cAMP-elevating agent. Forskolin was dissolved in DMSO (Sigma-Aldrich, Saint Louis, MO, USA) to a concentration of 60 μM, and cultures were incubated with this solution for 20 minutes. SQ22536 (Sigma-Aldrich, Saint Louis, MO, USA), an adenylyl cyclase inhibitor, was used to block the activity of adenylyl cyclase. SQ22536 was dissolved in DMSO to a final concentration of 100 μM, and the cultures were incubated with this solution for 15 minutes.

### Real-time Reverse Transcription-polymerase Chain Reaction (RT-PCR)

Cells were harvested and rinsed twice with 1X PBS. Total RNA was extracted using TRIzol (GIBCO-BRL, Rockville, MD, USA), and the concentration of the obtained RNA was quantified by measuring the absorbance with a spectrophotometer (ND-1000, NanoDrop, Wilmington, DE, USA) at 260 nm and 280 nm. First-strand cDNA was reverse-transcribed from 1 μg of RNA using Moloney murine leukemia virus reverse transcriptase and oligo-dT primers. Quantitative real-time PCR was performed in a Bio-Rad iQ5 real-time detection system (Bio-Rad Laboratories Inc, Hercules, CA), and the reactions were carried out in a 12.5 μl mixture containing cDNA, forward and reverse primers for each gene and SYBR® Green Real-time PCR Master Mix (Toyobo CO.,LTD. OSAKA JANPAN). Primer pairs for the following human genes were used: *GAPDH* (5′-GAA GGT GAA GGT CGG AGT CA-3′ forward and 5′-GAA GAT GGT GAT GGG ATT TC-3′ reverse), *TLR1* (5′-GTT CCT GGC AAG AGC ATT GT-3′ forward and 5′-ATG GGT TCC AGC AAG ATC AG-3′ reverse), *TLR2* (5′-GCA TGT GCT GTG CTC TGT TC-3′ forward and 5′-TTC TCC ACC CAG TAG GCA TC-3′ reverse), *TLR3* (5′-AGT GCC CCC TTT GAACTC TT-3′ forward and 5′-TCC CAG ACC CAA TCC TTA TC-3′ reverse), *TLR4* (5′-AAG CCG AAA GGT GAT TGT TG-3′ forward and 5′-TCC TCC CAC TCC AGG TAA GT-3′ reverse), *TLR6* (5′-AGT AGC TGG GCT TGC ATT GT-3′ forward and 5′-TTA TTG GAG GGC CTT GAG TG-3′ reverse), *Cox-2* (5′-TGA GCA TCT ACG GTT TGC TG-3′ forward and 5′-TGC TTG TCT GGA ACA ACT GC-3′ reverse), *IL-1β* (5′-GAC CTT CCA GGA GAA TGA CC-3′ forward and 5′-CAA AGG ACA TGG AGA ACA CC-3′ reverse), *IL-6* (5′-CTA GAG TAC CTC CAG AAC AG-3′ forward and 5′-TGA CCA GAA GAA GGA ATG C-3′ reverse) and *IL-8* (5′-TGC AGC TCT GTG TGA AGG TG-3′ forward and 5′-AAT TTC TGT GTT GGC GCA GT-3′ reverse). Relative mRNA expression levels were calculated from the threshold cycle (Ct) value of each PCR product and normalized to the housekeeping gene GAPDH using the comparative Ct method.

### Enzyme-linked Immunosorbent Assay (ELISA)

The supernatants of the cell cultures were collected to evaluate the concentrations of IL-6 and IL-8 using the Human IL-6 ELISA Ready-SET-Go Kit (Bioscience, Inc., Allentown, PA, USA) and the Human IL-8 ELISA Ready-SET-Go Kit (Bioscience, Inc., Allentown, PA, USA), respectively. Both kits were used according to the manufacturer’s instructions. The levels of IL-6 and IL-8 proteins were quantified at 450 nm using an ELISA plate reader. In addition, a Cyclic AMP EIA Kit (Cayman Chemical Company, Ann Arbor, MI, USA) was used to measure the generation of cyclic adenosine monophosphate (cAMP) within cells by collecting cell culture extracts. All procedures were based on the manufacturer’s instructions, and the plates were read at a wavelength of 450 nm.

### Western Blotting

Cells were washed twice in PBS and lysed using CelLytic™ M cell lysis reagent (Sigma-Aldrich, Saint Louis, MO, USA) with protease inhibitor (Complete Protease Inhibitor Cocktail Tablets; Roche Diagnostics Ltd. Burgess Hill, United Kingdom). Protein concentrations were determined using the BCA protein assay kit (Novagen, Darmstadt, Germany) based on the manufacturer’s instructions. Cell lysate containing 40 µg of proteins were analyzed by 8% SDS-Polyacrylamide gel electrophoresis. Transferred membranes were blocked using 5% non-fat milk and incubated overnight with antibodies against Phospho-NF-κB p65 (Ser536) (dilution 1∶1,000, Cell Signaling Technology, Inc., Beverly, MA, USA), NF-κB p65 (dilution 1∶6,000, Epitomics, Inc., Burlingame, CA, USA), IκBα Phospho (dilution 1∶2,000, Epitomics, Inc., Burlingame, CA, USA) and IκBα (dilution 1∶2,000, Epitomics, Inc., Burlingame, CA, USA). The same membrane was probed with anti-β-actin (dilution 1∶4,000) as a loading control. Finally, membranes were detected using the Western Lighting™ Chemiluminescence Reagent Plus (Perkin-Elmer, Boston, MA, USA). Blotted bands were digitally measured by using the UVP AutoChemi™ Image and Analysis System (UVP, Upland, CA, USA).

### Immunofluorescence staining

Cells were fixed with 4% paraformaldehyde (PANREAC QUIMICA, Castellar del Vallès, Spain) for 1 hour. After being washed, cells were permeabilized with 0.1% Triton X-100 for 10 min, blocked in 1% BSA for 1 hour, and immunostained with NF-κB p65 (Epitomics, Inc., Burlingame, CA, USA) at 4°C overnight. Cells were then incubated with Alexa Fluor 594-labeled goat secondary antibodies (Invitrogen, Carlsbad, CA, USA) for 1 hour. Finally, cell nuclei were stained with DAPI (Thermo SCIENTIFIC, Madison, WI, USA) and mounting on a coverslip with 50% glycerol in PBS. Fluorescence images were visualized using Nikon TE300 microscope and created by Image-Pro Plus 5.0 (MediaCybernetics, Silver Spring, MD, USA).

### Dual-luciferase Assay

Cells were co-transfected with the pGL 4.32[luc2P/NF-κB-RE/Hygro] vector (1 µg) (Promega, Madison, WI, USA) and pTK-Renilla vector (20 ng) (Promega, Madison, WI, USA). Electro-transfection was performed using a MicroPorator (Digital Bio Technology, South Korea) based on the manufacturer’s instructions. After 5 hours, luciferase activity was initiated using a Dual-Luciferase® Reporter Assay System (Promega, Madison, WI, USA) and measured with TopCount Microplate Scintillation and Luminescence Counters (Packard Biosciences, Waltham, MA, USA).

### Statistical Analysis

SPSS version 17.0 was used for statistical analysis. The results are expressed as the mean ± standard deviation. Statistically significant differences were determined using analysis of variance (ANOVA) followed by a post hoc Tukey’s test for multiple comparisons. A p-value of less than 0.05 was considered statistically significant.

## Results

### Determination of the Activity and Expression Pattern of TLRs in hADSCs

**Figure 1:**
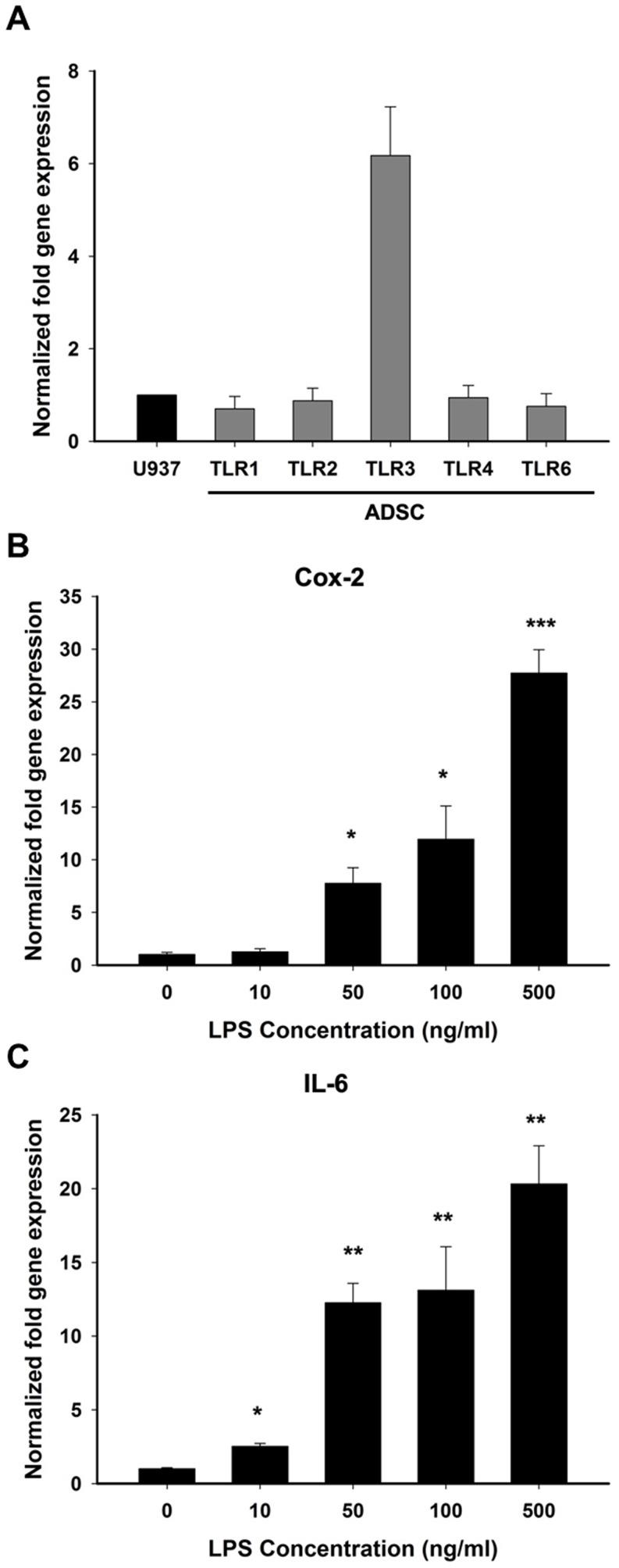
hADSCs expresses TLRs and stimulated by LPS. (**A**) The mRNA expression levels of all TLRs in the hADSCs were compared to the expression levels in U937 cells, as determined by real-time RT-PCR analysis; TLR3 showed a higher mRNA expression level in hADSCs than in U937 cells. (**B, C**) Determination of the optimal LPS treatment concentration by evaluating the expression of *Cox-2* and *IL-6* mRNA levels. The data are shown as the mean ± SD (n = 3). The following statistical levels were applied: *p<0.05, **p<0.01, and ***p<0.001 compared to the control.

To understand whether hADSCs can detect multiple pathogen-associated molecular patterns (PAMPs) via TLRs, we first had to ascertain the expression pattern of TLRs in hADSCs. Primers specific to TLR1, TLR2, TLR3, TLR4, and TLR6 were designed to analyze the basal mRNA expression levels of TLRs in hADSCs. The TLR expression levels in the hADSCs were compared to those of U937 cells, which express various TLRs [17]. The mRNA expression levels of all TLRs in the hADSCs except for TLR3 were similar to the expression levels in U937 cells, as determined by real-time RT-PCR analysis; TLR3 showed a higher mRNA expression level in hADSCs than in U937 cells (Fig. 1A).

Next, we analyzed the activity of TLRs in hADSCs. LPS, the ligand of TLR4, was used as a bacterial model in this study. hADSCs were treated with different concentrations of LPS, and the expression of *Cox-2* and *IL-6* mRNA was then evaluated. A dose-dependent increase in *Cox-2* and *IL-6* mRNA expression was observed 24 hours after LPS treatment (Fig. 1B, C). The levels of *Cox-2* and *IL-6* mRNA expression were significantly elevated when a concentration of LPS higher than 50 ng/ml was used (p<0.05 in *Cox-2* and p<0.01 in *IL-6*). These results indicated that hADSCs express TLRs and exhibit a functional TLR4. Additionally, treatment with LPS at a concentration of 50 ng/ml is sufficient to stimulate the production of pro-inflammatory mediators to initiate the microglial inflammation process.

### LPLI Decreases LPS-induced Pro-inflammatory Cytokine Gene Expression

**Figure 2:**
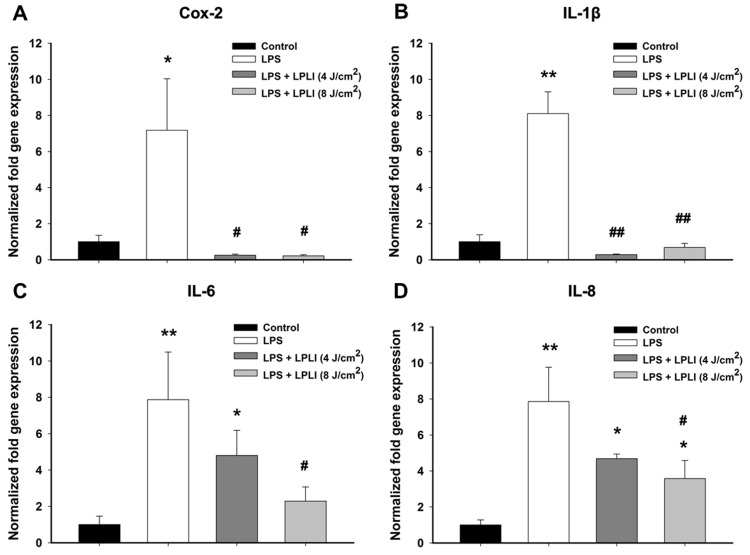
LPLI suppresses gene expression of pro-inflammatory cytokines by real-time RT-PCR. (**A**) *Cox-2.* (**B**) *IL-1β.* (**C**) *IL-6.* (**D**) *IL-8.* The results were analyzed using the 2^−ΔCT^ method based on the control. The data are shown as the mean ± SD (n = 6). The following statistical levels were applied: *p<0.05 and **p<0.01 compared to the control and #p<0.05 and ##p<0.01 compared to LPS (50 ng/ml).

hADSCs were treated with or without LPS (50 ng/ml), immediately followed by laser irradiation at energy densities of 4 J/cm^2^ or 8 J/cm^2^ to examine the effect of LPLI on the LPS-induced inflammatory response in hADSCs. The mRNA expression levels of pro-inflammatory cytokines, such as *Cox-2*, *IL-1β*, *IL-6*, and *IL-8,* were analyzed after 24 hours using real-time RT-PCR. LPS treatment strongly induced the expression of pro-inflammatory genes. However, when hADSCs were treated with a combination of LPLI and LPS, the mRNA levels of *Cox-2* and *IL-1β* were dramatically decreased, regardless of the LPLI energy density applied to the hADSCs (Fig. 2A, B). LPLI significantly reduced the levels of *IL-6* and *IL-8* mRNA expression in a dose-dependent manner (Fig. 2C, D); the *IL-6* and *IL-8* mRNA expression of group treated with energy density of 8 J/cm^2^ was significantly lower than the group that was only treated with LPS.

**Figure 3:**
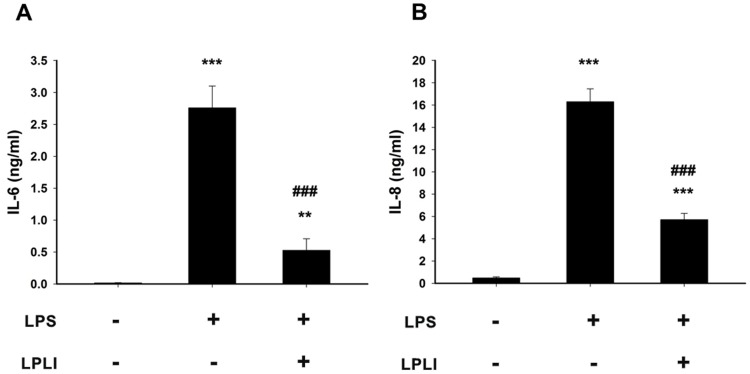
LPLI inhibits protein secretion of IL-6 and IL-8. The levels of pro-inflammatory proteins in LPS-stimulated hADSC culture media at 24 hours were determined by ELISA. (**A**) IL-6. (**B**) IL-8. The data are shown as the mean ± SD (n = 8). The following statistical levels were applied: **p<0.01 and ***p<0.001 compared to the control and ###p<0.001 compared to LPS (50 ng/ml).

To further confirm the effects of LPLI on the suppression of LPS-induced pro-inflammatory cytokines in hADSCs, the amounts of IL-6 and IL-8 secreted into the culture medium were measured using specific ELISAs. The concentrations of IL-6 and IL-8 showed similar trends of significant increases in the culture medium after 24 hours of LPS treatment. Co-treatment with LPLI (8 J/cm^2^) and LPS significantly suppressed IL-6 and IL-8 production (Fig. 3A, B). These results showed that LPLI significantly reduced the expression of LPS-induced pro-inflammatory genes at both the transcriptional and translational levels. However, the strength of the suppression effect generated by LPLI varied among different pro-inflammatory genes; this difference indicated that the regulatory mechanisms induced by LPLI affecting these genes might differ depending on the gene. In addition, we also used the lactate dehydrogenase (LDH) assay to determine whether LPLI induced cytotoxic effects on hADSCs. It showed that LPLI treatments did not induce significant cytotoxic effects on hADSCs (Fig. S1). The data indicated that LPLI reduced LPS-induced inflammatory response was not caused by harmful effects of LPLI.

### LPLI Downregulates LPS-induced NF-κB Activation

It has been shown that NF-κB is an important regulator in the LPS-induced inflammatory response [18], Thus, we investigated whether LPLI decreases the expression of LPS-induced pro-inflammatory cytokines by regulating NF-κB activity.

**Figure 4:**
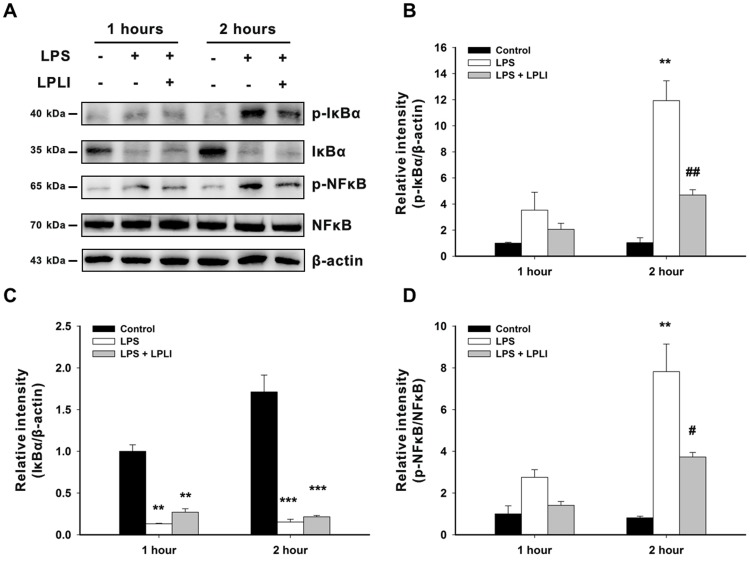
LPLI decreases IκBα degradation and NFκB activation. (**A**) hADSCs were treated with or without LPS (50 ng/ml), immediately followed by LPLI (8 J/cm^2^). After 1 hour and 2 hours, the protein expression of p-IκBα, IκBα, p-NFκB and NFκB was determined using Western blot analysis, and β-actin served as loading control. The blots were quantified, and the results were expressed as ratios compared to LPS (0 ng/ml) at 1 hour, which was defined as 1. (**B**) p-IκBα vs. β-actin. (**C**) IκBα vs. β-actin. (**D**) p-NFκB vs. NFκB. The data are shown as the mean ± SD of 3 independent experiments. The following statistical levels were applied: **p<0.01 and ***p<0.001 compared to the control and #p<0.05 and ##p<0.01 compared to LPS (50 ng/ml).

hADSCs were treated with or without LPS (50 ng/ml), immediately followed by LPLI (8 J/cm^2^). Treatment with LPS induced a slight increase in IκBα phosphorylation at 1 hour post-incubation and strongly increased IκBα phosphorylation at 2 hours post-incubation. In addition to the increases in p-IkBα expression, IKBα degradation was observed both 1 hour and 2 hours post-incubation (Fig. 4A–C). Treatment with LPS induced a 2.75-fold increase in the phosphorylation of NF-κB (p-NF-κB/NF-κB) at 1 hour post-incubation and a 7.82-fold increase at 2 hours post-incubation, which is linked to enhancement of NF-κB activation (Fig. 4A,D). However, when hADSCs were co-treated with LPS and LPLI, the expression of p-IκBα and p-NF-κB was inhibited at these two time points (Fig. 4A, B and D). Although our data showed that co-treatment with LPS and LPLI induced a 1.41-fold increase in NF-κB phosphorylation at 1 hour post-incubation and a 3.72-fold increase at 2 hours post-incubation, these increases are much lower than those seen following LPS treatment alone (Fig. 4D).

**Figure 5:**
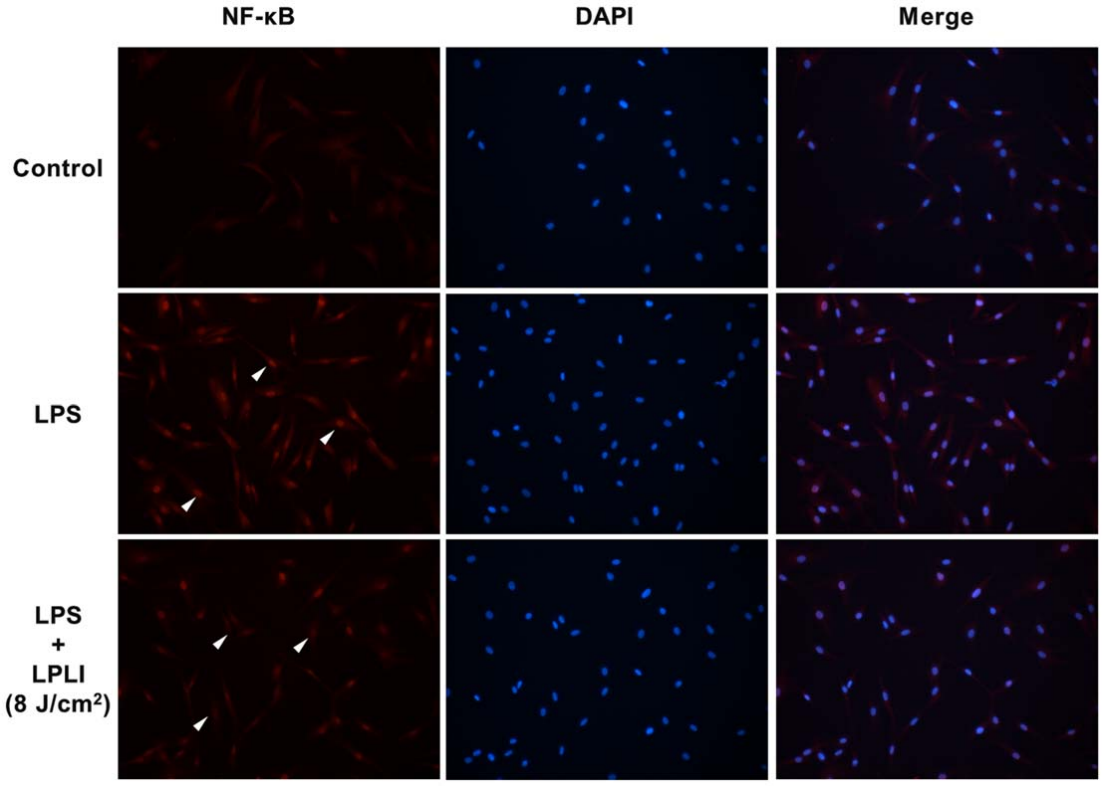
LPLI decrease nuclear translocation of NF-κB. hADSCs were pre-treated with or without LPS (50 ng/ml), followed by LPLI (8 J/cm^2^), and the localization of the NF-κB was determined by fluorescence. The images in the upper panel show the cytoplasmic localization of NF-κB in the control cells. The image in the middle panel shows the nuclear translocation of NF-κB in cells treated with LPS. The image in the lower panel shows that LPLI treatment blocked the nuclear translocation of NF-κB caused by LPS stimulation.

**Figure 6:**
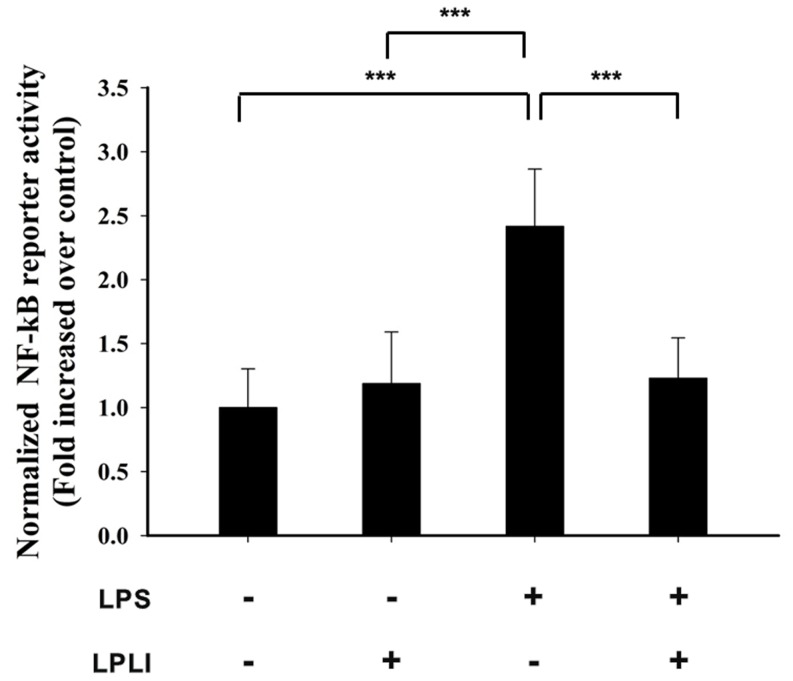
LPLI decreases the transcription activity of NFκB. hADSCs were transfected with the vectors pGL-NFκB and pTK-Renilla (control vector), and the cells were then incubated with 50 ng/ml LPS with or without LPLI. Luciferase activity was assayed 5 hours after LPLI treatment. The data are expressed as the mean ± SD of 3 independent experiments. The statistical level applied was ***p<0.001.

Phosphorylation and nuclear translocation of NF-κB are required for NF-κB activation. We monitored the cellular distribution of NF-κB using fluorescence microscopy and found that LPLI blocked the nuclear accumulation of NF-κB resulting from LPS stimulation (Fig. 5). The transcriptional activity of NF-κB was determined by measuring relative luciferase activity. Treatment of hADSCs with LPLI alone did not affect luciferase activity, whereas treatment with LPS caused a 2.5-fold increase in luciferase activity. Luciferase activity was significantly inhibited following co-treatment of hADSCs with LPLI and LPS (Fig. 6). Together, these results indicated that LPLI suppressed pro-inflammatory cytokine expression through inhibition of NF-κB activation.

### LPLI Modulated NF-κB via the cAMP Signaling Pathway

**Figure 7:**
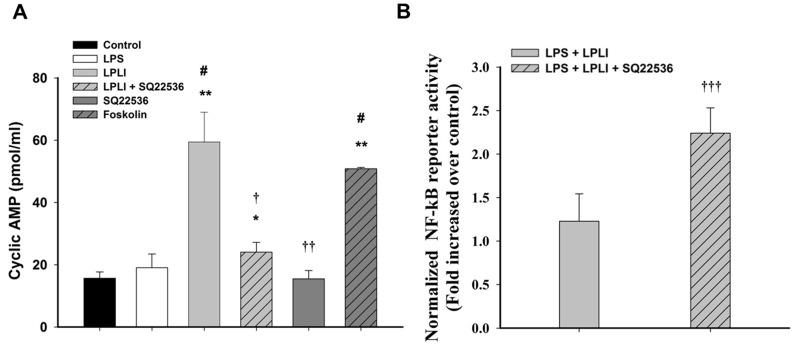
LPLI increases intracellular cAMP level to suppress NF-κB transcriptional activity. (**A**) Intracellular cAMP levels were measured by ELISA in hADSCs treated with LPS, LPLI, SQ22536, and forskolin. (**B**) NF-κB transcriptional activity was assayed 5 hours after LPLI treatment only and combined pretreatment of SQ22536. The data are expressed as the mean ± SD (n = 6). The following statistical levels were applied: *p<0.05 and **p<0.01 compared to the control, #p<0.05 compared to LPS (50 ng/ml), and †p<0.05, ††p<0.01, and †††p<0.001 compared to LPLI (8 J/cm^2^).

**Figure 8:**
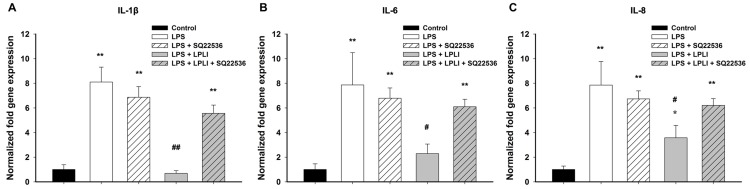
SQ22536 blocks the anti-inflammatory response of LPLI by real-time RT-PCR. (**A**) *IL-1β.* (**B**) *IL-6.* (**C**) *IL-8.* The results were analyzed by the 2^−ΔCT^ method based on the control. The data are shown as the mean ± SD (n = 6). The following statistical levels were applied: *p<0.05 and **p<0.01 compared to the control and #p<0.05 and ##p<0.01 compared to LPS (50 ng/ml).

cAMP acts not only as a modulator of immune function but also as a regulator of LPLI signaling [19]. Thus, we determined the cAMP levels associated with LPLI-mediated anti-inflammatory activity in hADSCs. Treatment of hADSCs with either LPS or SQ22536, an adenylyl cyclase inhibitor, did not alter the level of cAMP, whereas the adenylyl cyclase activator forskolin significantly increased cAMP accumulation (Fig. 7A). A similar result was obtained by treating hADSCs with LPLI, which resulted in significant elevation of the cAMP concentration. This LPLI-mediated cAMP accumulation was blocked by pretreatment with SQ22536 (Fig. 7A). To investigate whether LPLI-mediated cAMP accumulation is the crucial mechanism involved in decreasing pro-inflammatory cytokine expression in hADSCs, we first determined whether cAMP signaling regulates the NF-κB activity using luciferase reporter assay. We found that SQ22536 pretreatment blocked the inhibitory effect of LPLI-mediated NF-κB transcriptional activity in LPS treated hADSCs (Fig. 7B). Next, we measured the mRNA expression levels of pro-inflammatory cytokines (IL-1, IL-6, and IL-8) in hADSCs treated with LPS or LPLI with or without SQ22536 pretreatment. Following pretreatment with SQ22536, the LPLI-mediated inhibition of pro-inflammatory cytokine expression was completely reversed. However, SQ22536 did not affect the mRNA expression of pro-inflammatory cytokines (IL-1, IL-6, and IL-8) induced by LPS (Fig. 8A–C). These results indicated that the LPLI-mediated anti-inflammatory activity observed in hADSCs might occur through the cAMP signaling pathway.

## Discussion

The results of the present study show for the first time that LPLI (660 nm) suppresses the inflammatory reaction induced by LPS in hADSCs. We first confirmed that hADSCs exhibit a similar expression profile of TLRs compared with U937 cells, a human leukemic monocyte lymphoma cell line. Our results correspond with those of other recent studies. Cho et al. reported that hADSCs showed intense expression of TLR2, TLR3, TLR4, and TLR6 and lower expression of TLR1, TLR5 and TLR9 [20]. Lombardo et al. showed that hADSCs express TLR1 through TLR10, with the exception of TLR8 [21]. Together, these data indicated that hADSCs express TLRs and are able to respond to various TLR ligands associated with inflammation.

In the past few years, a number of findings have indicated that LPLI suppresses the inflammatory reaction both in vitro and in vivo. Correa et al. used LPS to induce peritonitis in mice and found that LPLI (GaAs laser, 904 nm) can reduce inflammatory cell migration in a dose-dependent manner, with an energy dose of 3 J/cm^2^ being the most effective [22]. Pires et al. found that LPLI (780 nm), at an energy dose of 7.7 J/cm^2^, decreased the expression of IL-6, COX-2, and TGF-β associated with collagenase-induced tendinitis [23]. Boschi et al. showed that LPLI (InGaAlP laser, 660 nm) significantly reduced the expression of NO, IL-6, MCP-1, IL-10, and TNFα [24]. In LPS challenged hADSC, we obtained similar results to those observed in the aforementioned studies, finding that LPLI significantly inhibited the mRNA expression of pro-inflammatory cytokines (Cox-2, IL-1, IL-6, and IL-8) and secreted proteins (IL-6 and IL-8), leading us to conclude that LPLI has an anti-inflammatory effect in hADSCs.

It is well known that NF-kB is a crucial transcription factor involved in the regulation of inflammation. Our data showed that LPLI suppressed the expression of pro-inflammatory cytokine genes. Therefore, we focused on the relationship between LPLI and the activity of NF-κB. The classic NF-κB is a heterodimer composed of class I (p50, p52) and class II proteins (Rel-A/p65, Rel-B, and c-Rel). In the cytoplasm of unstimulated cells, NF-κB forms a complex with inhibitors of NF-κB (IκBs). After stimulation by upstream signals, the IκB kinase (IKK) complex phosphorylates IκBs, leading to proteasome-mediated degradation and dissociation of IκBα and NF-κB [25]. Next, nuclear translocation of NF-κB occurs and induces gene transcription; in this process, the phosphorylation state of NF-κB has been linked to enhancement of NF-κB activation. We observed that LPLI decreased the level of phosphorylated IκBα and maintained more inactive IκBα in the cytoplasm. LPLI also inhibited the LPS-induced expression of phospho-NF-κB and clearly decreased the amount of NF-κB that was translocated. Furthermore, LPLI significantly inhibited the transcriptional activity of NF-κB. Results similar to ours were also reported by Aimbire et al [26], who showed that a low-level diode laser (660 nm) at an energy density of 7.5 J/cm^2^ reduced the mRNA levels of both Bcl-xL and A1, which are produced by NF-κB nuclear translocation, leading these authors to suggest that the anti-inflammatory effect of LPLI can be mediated by NF-κB. Rizzi et al. indicated that LPLI (904 nm) at an energy density of 5 J/cm^2^ was able to block the degradation of IkBα, which is consistent with our findings [27]. Together, these data suggest that LPLI inhibits the inflammatory process via inhibition of NF-κB transcriptional activity.

Several studies have shown that the intracellular levels of cAMP are increased by LPLI [28], [29]. cAMP is an important second messenger in many biological processes, such as cell proliferation, differentiation, apoptosis, and inflammation, that is produced by the activation of adenylyl cyclases and converted from ATP [30]. Recent data have shown that elevation of intracellular cAMP levels inhibits the transcriptional activity of NF-κB [31], [32]. Possible mechanisms underlying the role of cAMP in regulating NF-κB activity include the ability of cAMP to manage Iκκ activity and IκB degradation as well as to change the composition of NF-κB dimers and thereby block transcription [33]. In the present study, we observed that the level of cAMP increased by approximately 3–4 fold following LPLI treatment. This finding indicated that LPLI can act to stimulate the level of cAMP. To confirm that the inhibition of inflammation by LPLI occurred via cAMP, we treated hADSCs with the adenylyl cyclase inhibitor SQ22536 and observed that the inhibitory effect by LPLI was dramatically reduced. Overall, our results indicated that cAMP is an important mediator involved in the inhibitory effect of LPLI on LPS-induced inflammation in hADSCs.

In conclusion, our results show that hADSCs express TLR1, TLR2, TLR3, TLR4, and TLR6. Following LPS (50 ng/ml) stimulation, hADSCs exhibited a significant increase in the production of pro-inflammatory mediators (Cox-2, Il-1β, IL-6, and IL-8). The major findings of the present study are that low power laser irradiation markedly inhibited the LPS-mediated inflammatory response in hADSCs at an optimal dose of 8 J/cm^2^. The inhibitory effect stimulated by LPLI might act via increasing the intracellular level of cAMP, resulting in down-regulation of NF-κB transcriptional activity. Our results indicate that LPLI treatment can potentially be applied in anti-inflammatory therapy followed by stem cell therapy.

## Supporting Information

Figure S1 **LPLI treatments did not induce cytotoxic effects on hADSCs.** hADSCs were treated with LPLI at doses of 0 (control), 4, or 8 J/cm^2^. LDH leakage was analyzed to evaluate cell cytotoxicity at 24 hours. There were no significant differences between the groups (n = 12).
(TIF)
