# Supplementary figures and images for: Low-Power Laser Irradiation Suppresses Inflammatory Response of Human Adipose-Derived Stem Cells by Modulating Intracellular Cyclic AMP Level and NF-κB Activity
Source: PLoS One. 2013 Jan 16;8(1):e54067. doi: 10.1371/journal.pone.0054067 (PMC3546978; doi:10.1371/journal.pone.0054067)

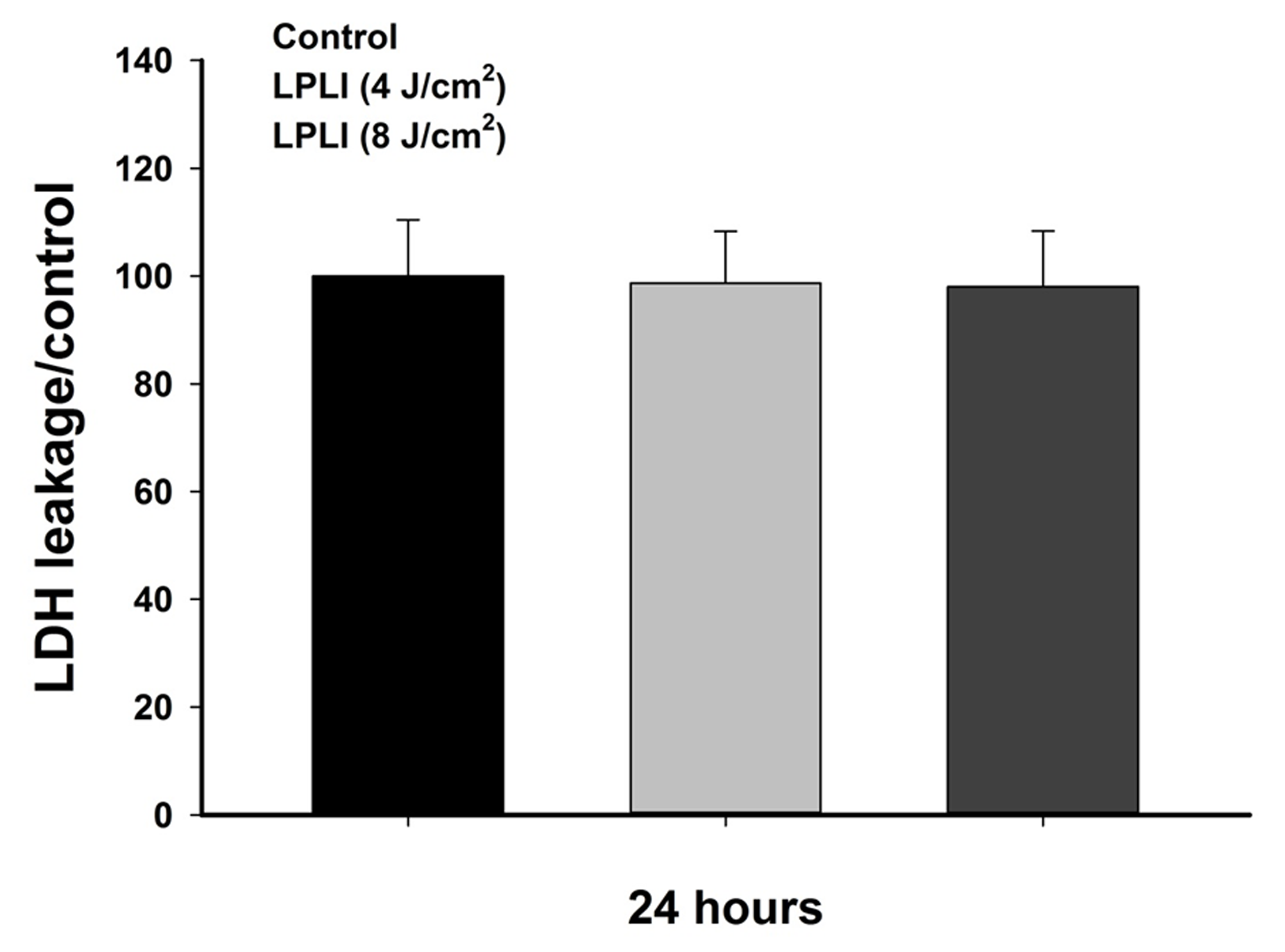

Supplement: Figure S1 — LPLI treatments did not induce cytotoxic effects on hADSCs. hADSCs were treated with LPLI at doses of 0 (control), 4, or 8 J/cm2. LDH leakage was analyzed to evaluate cell cytotoxicity at 24 hours. There were no significant differences between the groups (n = 12). (TIF) [file pone.0054067.s001.tif]
